# Bread Sourdough Lactic Acid Bacteria—Technological, Antimicrobial, Toxin-Degrading, Immune System-, and Faecal Microbiota-Modelling Biological Agents for the Preparation of Food, Nutraceuticals and Feed

**DOI:** 10.3390/foods11030452

**Published:** 2022-02-03

**Authors:** Elena Bartkiene, Fatih Özogul, João Miguel Rocha

**Affiliations:** 1 Department of Food Safety and Quality, Lithuanian University of Health Sciences, Tilzes 18, LT-47181 Kaunas, Lithuania; 2 Institute of Animal Rearing Technologies, Lithuanian University of Health Sciences, Tilzes 18, LT-47181 Kaunas, Lithuania; 3 Department of Fishing Technology and Seafood Processing Technology, Faculty of Fisheries, Cukurova University, Balcalı, Adana 01330, Turkey; fozogul@cu.edu.tr; 4 LEPABE—Laboratory for Process Engineering, Environment, Biotechnology and Energy, Faculty of Engineering, University of Porto, Rua Dr. Roberto Frias, 4200-465 Porto, Portugal; jmfrocha@fe.up.pt; 5 ALiCE—Associate Laboratory in Chemical Engineering, Faculty of Engineering, University of Porto, Rua Dr. Roberto Frias, 4200-465 Porto, Portugal

**Keywords:** sourdough, lactic acid bacteria, food, feed, nutraceuticals, antimicrobials

## Abstract

This review intends to highlight the fact that bread sourdough is a very promising source of technological, antimicrobial, toxin-degrading, immune system-, and faecal microbiota-modelling biological agents for the preparation of food, nutraceuticals, and feed, which has great potential at industrial biotechnology scale. There are many applications of sourdough lactic acid bacteria (LAB), which are the main microorganisms in spontaneous sourdough. In addition to their application as pure technological strains in the food and feed industries, taking into consideration the specific properties of these microorganisms (antimicrobial, antifungal, immuno-, and microbiota-modulating, etc.), they are used as valuable ingredients in higher-value food as well as nutraceutical formulations. Additionally, a very promising application of LAB is their use in combination with plant- and/or animal-based ingredients to increase the functional properties of the whole combination due to different mechanisms of action, as well as desirable symbiotic activity. In addition to traditional foods prepared using sourdough microorganisms (bread, biscuits, meat products, dairy, beverages, etc.), they could find application in the preparation of added-value ingredients for the food, nutraceutical, and feed industries. Finally, this mini-review gives a brief introduction to the possible applications of sourdough LAB in the food, feed, and nutraceutical industries.

## 1. Introduction

Bread sourdough is lacto-fermented flour with acidic properties, which shows potential to improve the technological properties of milled cereals. The fermentation process can be applied for bread flour (wheat and rye) as well as for non-gluten and non-bread flours (pea, lupine, soya, etc.). Sourdough preparation and the cereal fermentation process is the most important part of processing, almost for any cereal [1,2]. Spontaneous fermentation is the oldest process of sourdough preparation. Despite control of this process being difficult, till now, spontaneous sourdough preparation has been used in small bakeries. Typically, sourdough contains two main ingredients, flour and water, which, during microbial bioconversion, develop sour characteristics. The main microorganisms in sourdough are lactic acid bacteria (LAB). In sourdough, various yeasts (especially species of the genera *Saccharomyces* and *Candida*) are also active in symbiosis with LAB. The main metabolites of sourdough microbial conversion are organic acids, carbon dioxide, diacetyl, and alcohols [3]. However, spontaneous fermentation of cereals that are contaminated with undesirable microorganisms can lead to low product quality as well as hazards. It has been reported that various species of microorganisms can start to dominate in spontaneous cereal fermentations. However, microorganisms belonging to the genus *Lactobacillus* are usually predominant in sourdoughs [4], and from this point of view, spontaneously fermented cereals are good source of LAB and various yeast species [5]. However, the predominant microflora and their characteristics in fermented cereals are related to many factors, including geographical location, processing, handling, etc. Predominant LAB strains in Lithuanian sourdough were *Lactobacillus plantarum*, *Lactobacillus casei*, *Lactobacillus curvatus*, *Pediococcus pentosaceus*, *Pediococcus acidilactici*, *Lactobacillus paracasei*, *Lactobacillus brevis*, and *Lactobacillus uvarum* [6]. Additionally, the microbial community developing in the sourdough may be formed in relation to flour and water contamination, the starters used, environmental factors (contamination of fermentation vessel, air, etc.), etc. [7,8,9,10,11]. LAB include several genera: *Lactobacillus*, *Lactococcus*, *Leuconostoc*, *Oenococcus*, *Pediococcus*, *Streptococcus*, *Tetragenococcus*, *Aerococcus*, *Carnobacterium*, *Enterococcus*, *Vagococcus*, and *Weissella* [12,13]. Despite the genera *Lactobacillus* and *Saccharomyces* being reported as the most dominant microorganisms in sourdough, their characteristics and properties (technological, antimicrobial, toxin-degrading, etc.) can vary from species to species. Therefore, isolation, characterisation, and identification of the microorganisms involved in spontaneous cereal fermentation, with prospective selection of starter cultures for various purposes, has become very interesting.

**Figure 1:**
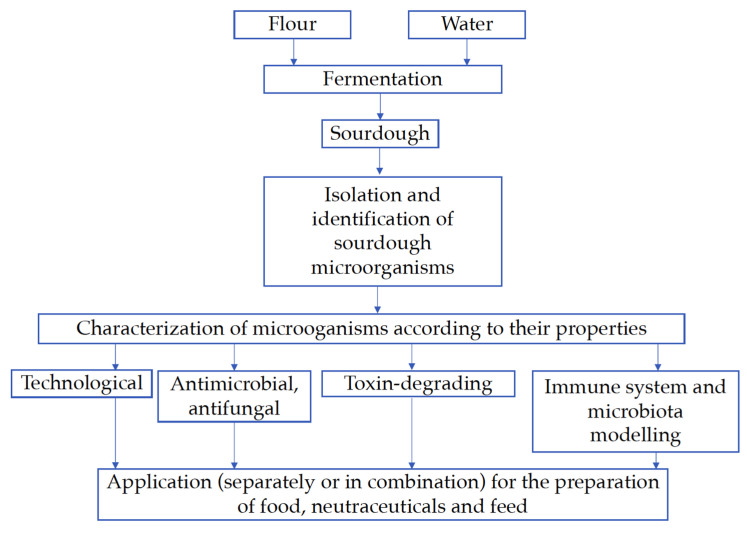
Schematic representation of the brief introduction of this review.

There are many applications of sourdough LAB and their metabolites in food and feed biotechnology. Due to the specific properties of these microorganisms (antimicrobial, antifungal, immuno- and microbiota-modulating, etc.), they are used as valuable ingredients in nutraceutical formulations [6]. A very promising application of LAB is their use in combination with plant- and/or animal-based ingredients to increase the functional properties of the whole combination due to different mechanisms of action, as well as desirable higher antimicrobial activity [14,15]. In particular, antimicrobial activity of combinations based on microbial-, plant-, and/or animal-based ingredients is of interest because it can lead to a reduction in the dose of each compound used in antimicrobial formulations [16]. This finding is very important because there are global problems with resistance of pathogens to antimicrobials, and LAB, which possess antimicrobial activity, could be valuable ingredients in natural antimicrobial combinations. In addition to traditional foods prepared using sourdough microorganisms (bread, biscuits, meat products, dairy, beverages, etc.), they could find application for the preparation of added-value ingredients for the food, nutraceutical, and feed industries. In particular, in the feed industry, probiotics application is very promising [17,18]. A schematic representation of the brief introduction of this review about sourdough LAB as technological, antimicrobial, toxin-degrading, and immune system- and digestive microbiota-modelling biological agents for preparation of food, nutraceuticals, and feed is shown in Figure 1.

## 2. Sourdough LAB—Technological Starters for Food Preparation

### 2.1. Pure Sourdough Starters and Their Combinations for Preparation of Higher-Value Safer Bread

**Figure 2:**
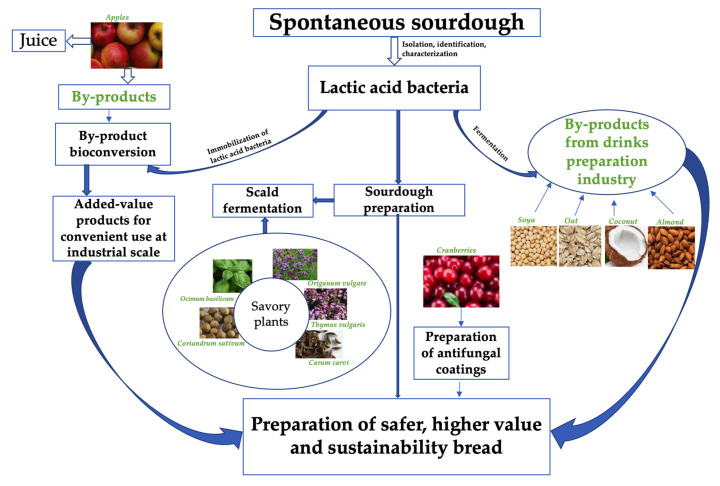
Possible uses of pure lactic acid bacteria starters and their combinations for the preparation of safer, higher-value, and sustainable bread.

A summary of the possible uses of pure LAB starters and their combinations in bread technology is illustrated in Figure 2.

As spontaneous fermentation cannot guarantee a stable process, starter cultures are more and more often involved in the bread production chain. In addition to increasing process stability, LAB starters can be used for fermenting different varieties of cereal. These can be incorporated in the main wheat, wheat–rye, and rye bread formulations to increase the nutritional value of the end product (the incorporation of non-bread cereal flour has especially big potential when wheat bread is produced from refined wheat flour). The potential use of *Pediococcus acidilactici* LUHS29 strain immobilised in apple pomace has been reported for barley sourdough fermentation and the preparation of higher-value bread [19]. The LUHS29 strain was selected for preparation of higher-value bread due to its versatile carbohydrate metabolism and good resistance to an acidic environment. Experiments showed that the LUHS29 strain increases lactic acid production, β-glucan solubility, and the total phenolic compound content, as well as radical scavenging activity in barley sourdough, in comparison with spontaneously fermented barley sourdough. Higher functionality and better technological properties in sourdough prepared with LUHS29 starter cultures lead to a lower acrylamide content in bread and reduce the bread staling process.

In addition to separate LAB strains being used for cereal fermentation, recently, attention has been paid to the use of LAB combinations in the production of bread in order to give the product exceptional properties. The possible use of combinations of LAB strains (*Pediococcus pentosaceus* LUHS183 and *Leuconostoc mesenteroides* LUHS242, *P. pentosaceus* LUHS183 and *Lactobacillus brevis* LUHS173, *P. pentosaceus* LUHS183 and *Enterococcus pseudoavium* LUHS234, *P. pentosaceus* LUHS183 and *Lactobacillus curvatus* LUHS51, *Lactobacillus plantarum* LUHS135 and *L. curvatus* LUHS51, *L. plantarum* LUHS135 and *P. pentosaceus* LUHS183) isolated and characterised from spontaneously fermented wheat sourdough for the preparation of wheat sourdough has been reported, and prepared sourdoughs were tested for wheat bread production [20]. Most of the tested LAB starters possessed versatile carbohydrate metabolism, and most of them were able to ferment L-arabinose, D-ribose, D-galactose, D-fructose, and D-maltose. Additionally, most of the selected LAB starters showed high tolerance to acidic conditions (high viability at pH 2.5 for 2 h). This characteristic is very important to ensure sourdough stability during processing. Different LAB combinations led to different bread sensory properties, and the highest overall acceptability was shown for bread samples prepared with sourdough fermented with the *L. plantarum* and *P. pentosaceus* combination. Despite this sourdough starter combination increasing the specific volume and porosity of bread samples, the lowest acrylamide concentration was found in bread fermented with the *P. pentosaceus* and *L. curvatus* combination (3.32 μg kg^−1^). Acrylamide content in bread is related to many factors, and the selection of LAB according to the bread formula should be taken into consideration. Primarily acrylamide formation in bread is related with the presents of precursors: reducing sugars and asparagine. In addition, proteolysis and acidification of dough can directly and indirectly affect both sugar and amino acid composition, and low pH and high total titratable acidity values in the dough inhibit acrylamide formation. Finally, LAB combinations showed great potential for increasing bread quality parameters and safety.

Despite bread being one of the most popular foods in the world, it is one of the most discarded because of its short shelf life (staling process) and sensitivity to mould spoilage [21]. To avoid bread waste, strategies to increase its shelf life are of great interest. One of the possibilities to prolong the shelf life of bread is to select starter cultures that possess not just good technological properties but also additional antifungal properties. Bartkiene et al. reported the antifungal effect and technological properties of *Lactobacillus coryniformis* LUHS71, *L. curvatus* LUHS51, *L. farraginis* LUHS206, and *Leuconostoc mesenteroides* LUHS225 strains [22]. The study showed that the incorporation of 15–20% sourdough increases the specific volume and porosity of bread and reduces the acrylamide concentration; surface treatment of bread with LUHS71, LUHS51, LUHS206, and LUHS225 strains immobilised in an apple by-product pomace prolonged the shelf life of bread. The antifungal activity of LAB is associated with the action of a combination of their excreted metabolites (acetic, propionic, caproic acids, etc.), in which caproic acid show a highest activity against moulds, which causes bread spoilage. Lactic, acetic, and phenyllactic acids in sourdough could significantly reduce bread spoilage caused by moulds. However, it should be pointed out that most of the metabolites’, including antifungal peptides, formation during fermentation is LAB species- and fermentable substrate-specific. In addition to sourdough LAB, antifungal compounds of plant origin can be attractive candidates for bread surface preservation against fungal spoilage. The major phenols of apple are benzoic acids, flavanols, terpenes, and derivatives such as norisoprenoids and coumaran, and most of them possess strong antimicrobial activity. Finally, there could be various applications of sourdough LAB in bread production: from sourdough starters to the preparation of antifungal coatings.

The concept of preventing mould spoilage and reducing acrylamide in wheat bread, using lactobacilli isolated from spontaneous sourdough in combination with a cranberry-based coating for bread surface treatment, has also been reported [23]. The antifungal activity of LAB strains *Pediococcus pentosaceus* LUHS183, *P. acidilactici* LUHS29, *Lactobacillus *paracasei** LUHS244, *Lactobacillus brevis* LUHS173, *Lactobacillus plantarum* LUHS135 and *Leuconostoc mesenteroides* LUHS242 strains, tested by the agar well diffusion assay, showed antifungal activity against *Aspergillus *nidulans**, *Penicillium funiculosum*, and *Fusarium poae.* Despite the tested LUHS173 and LUHS242 strains showing the weakest antifungal characteristics (in comparison with other tested LAB strains), they had the best technological and acrylamide-lowering properties. Finally, the tested approach showed antifungal activity against *Aspergillus fischeri*, *Penicillium oxalicum*, *P. funiculosum*, *F. poae*, *Alternaria alternata*, and *Fusarium graminearum*, and the authors stated that sourdough fermented with the selected LAB strains, in combination with an antimicrobial LAB–cranberry coating, could improve the quality and safety characteristics of bread as well as extend its shelf life.

Bread is the most popular food in the human diet; from this point of view, it is very important to ensure not only the main quality characteristics and biosafety of bread but also the chemical safety of this product. In addition to sourdough fermentation, scalded flour, before fermentation, could be enriched with savoury plants. The latter possesses antioxidant properties and can reduce acrylamide in bread. Bartkiene et al. [24] reported that the inclusion of savoury plants (*Thymus vulgaris*, *Carum carvi*, *Origanum* *vulgare*, *Ocimum basilicum*, and *Coriandrum sativum*) in the main bread formulation (in scalded flour) and fermentation with *Lactobacillus plantarum* LUHS135 significantly reduces the content of acrylamide in bread, especially using 5% scalded flour fermented with *L. plantarum* and supplemented with *O. vulgare* and 15% scalded flour fermented with *L. plantarum* and supplemented with *C. sativum*.

Sourdough LAB can also be applied for the fermentation of non-bread cereals to improve their technological properties before they are incorporated into the main bread formula [25]. This is a very promising solution for the valorisation of food industry by-products, which often have a high nutritional and functional value and contain specific compounds desirable for human nutrition (minerals, dietary fibre, proteins, vitamins, etc.). The valorisation of food industry by-products is beneficial for both the environment and the food industry. However, in most cases, the direct incorporation of by-products in the main food formula is complicated due to their specific technological properties, which are associated with reduced strength of the gluten network, which leads to bread with a lower volume and porosity. However, fermentation with selected LAB strains can lead to the valorisation of the whole by-product and, further, incorporation into the main bread formula. It has been reported that by-products from the preparation of almond, coconut, and oat drinks, ultrasonicated (at 37 kHz) and fermented with *Lacticaseibacillus casei* LUHS210, can be used for wheat bread enrichment; the highest overall acceptability was shown for bread prepared with 20% fermented almond drink by-product and 15% fermented oat drink by-product. Additionally, selected quantities of fermented by-products showed potential to reduce the staling process of bread during storage, and the lowest acrylamide content was found in the bread prepared with oat drink by-product. Finally, the authors recommended the incorporation of 15% fermented oat drink by-product to increase the overall acceptability of wheat bread and reduce its acrylamide content.

Until now, it has also been a big challenge to utilise okara (a by-product from soymilk production) [26]. However, the pre-treatment of okara by fermentation with selected LAB strains could be applied to maximise the health benefits of this by-product. Juodeikiene et al. [27] reported that okara treated with low-frequency ultrasound could be used for lacto-fermentation; the product obtained showed a high L-lactic acid concentration as well as a high LAB cell count, properties desirable for bread sourdough. By adding 5% ultrasonicated and fermented with *Lactobacillus paracasei* LUHS244 okara to the wheat bread formula, a higher porosity and specific volume of the bread were achieved, in comparison with untreated soya-based ingredients. Additionally, 5% ultrasonicated and fermented okara increased the overall acceptability of bread. Finally, ultrasonication and fermentation showed a potential improvement in the functionality of okara and the possibility of applying this by-product in the preparation of wheat bread.

Finally, it can be stated that the use of LAB starters isolated from spontaneous sourdough, with known characteristics, has a wide range of benefits, including not just safer, higher-value and sustainable bread preparation but also the reduction in challenges associated with the valorisation of food industry by-products (Table 1). Finally, fermentation technology and sourdough microbiota lead to a more environmentally friendly industry.

### 2.2. Challenges Associated with Lacto-Fermentation of Meat and Meat Products by Using Sourdough Lactic Acid Bacteria

**Figure 3:**
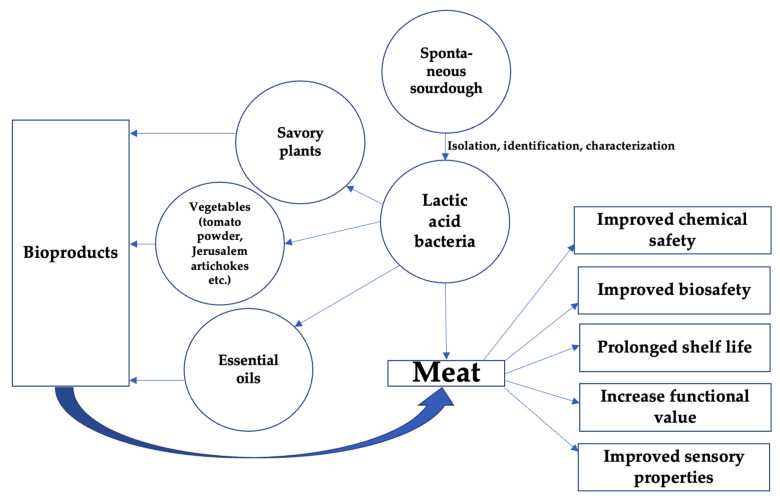
Possible uses of pure lactic acid bacteria starters and their combinations for the preparation of safer, higher-value, and more acceptable meat and meat products.

The possible uses of pure LAB starters and their combinations for the preparation of safer, higher-value, and more acceptable meat and meat products are shown in Figure 3.

Despite LAB being very popular as starters in meat fermentation, there are some challenges associated with the safety characteristics of fermented meat and meat products [28]. Due to the desirable antimicrobial properties of technological LAB starters, they improve biosafety, nutritional, and sensory characteristics, as well increasing the shelf life of fermented foods [29]. Additionally, the application of technological LAB starters can be associated with their biodegradation and/or the absorption of toxic compounds, i.e., polycyclic aromatic hydrocarbons (PAHs) and heterocyclic aromatic amines [30,31]. Despite all the above-mentioned benefits, fermented meat and meat products can be a source of undesirable compounds—biogenic amines (BAs) [32,33]. A low concentration of BAs can be found in most foods, as well as in mammals, and they are considered non-toxic; however, some BAs (putrescine and cadaverine) can form nitrosamines, which are carcinogenic, especially in meat and meat products, where nitrite is used as a technological compound. The main safety issue is that polyamines and diamines can form stable N-nitroso compounds [34]. In addition to the search for technological solutions, the issues of how to ensure the biosafety of meat and meat products and how to reduce BAs and PAHs, as well as control their concentration in meat and meat products, remain very important.

Finally, despite separate ingredients being safe (LAB have GRAS status; however, they can be associated with the formation of BAs in meat and meat products), the safety of the end product—fermented meat and meat products—must be taken into consideration because many changes can occur during the technological processes.

#### 2.2.1. Sourdough LAB for Reducing the Concentration of PAHs and BAs in Meat Products

Some toxic compounds are formed during the interaction of food ingredients, which occurs during traditional technological processes: drying, smoking, fermentation, etc. [35,36,37,38,39,40]. Among the toxic compounds in food, PAHs and BAs are the most prominent [41,42,43]. As exposure to these undesirable compounds is considered to have a negative influence on health problems, different methods have been developed to reduce the concentration of these substances in foods. In addition to toxin-lowering strategies, which include the elimination of and/or reduction in the content of precursors in raw materials, changes to technological process conditions, adding antioxidants, etc. [44,45,46,47], it has been reported that treatment with selected sourdough LAB can reduce the concentration of toxic substances in meat and meat products.

Recently, the reduction in PAHs in food by LAB has gained increasing attention because of their GRAS status; additionally, they are more acceptable to consumers, in comparison with chemical preservatives [48]. The physical adsorption mechanism by which LAB reduce PAHs in meat products has been reported [30,49,50], with the main binding site of benzo[a]pyrene being peptidoglycans on the cell wall [51]. Additionally, it has been reported that some LAB strains possess amine oxidase activity and can reduce the BA concentration in food [52,53,54]. Another mechanism of BA reduction in food is explained by the contribution of LAB to the inhibition of foodborne pathogens (i.e., *Staphylococcus aureus*, *E. coli*), which are associated with the production of BAs [33,55,56,57,58]. The cell-free supernatant of LAB can also reduce the concentration of BAs, and this mechanism of action is explained by the antibacterial substances and organic acids in the supernatant inhibiting the growth of undesirable BA-forming strains [59,60,61,62]. Sourdough LAB (in addition to inhibiting the pathogenic and opportunistic strains *Pseudomonas aeruginosa*, *Escherichia coli*, *Staphylococcus aureus*, *Salmonella enterica* serovar Typhimurium, *Bacillus cereus*, *Listeria monocytogenes*, *Yersinia enterocolitica*, and *Y. pseudotuberculosis*) can also contribute to the reduction in BAs and PAHs in cold smoked pork meat sausages [30]. Pre-treatment of sausage surfaces with the above-mentioned LAB strains isolated from bread sourdough, before smoking, decreases the concentration of cadaverine and spermidine in meat products. In addition, treatment after smoking reduces the content of putrescine (in the outer and centre layers of sausages). In the case of PAH concentration, pre-treatment (before or after smoking) of sausages with sourdough LAB significantly decreases the concentration of chrysene and benzo[a]pyrene in products. In addition to reducing toxic compounds, the use of LAB producing bacteriocin-like inhibitory substances can control technological parameters of the process such as fermentation microflora and indirectly reduce the formation of BAs in foods, which, in many cases, depends on the activity of the spoilage bacteria as well as of the technological starters, in which decarboxylase activity is high [30].

#### 2.2.2. Sourdough LAB-Based Marinades for Meat Pre-Treatment

The essential role of bacteria, yeasts, and moulds in spontaneous meat fermentation was known early in the mid-nineteenth century [63]. This is a useful technology to increase the water-holding capacity, tenderness, and flavour of meat and meat products [64]. It has been reported that a natural marinade, based on LAB strains isolated from spontaneous sourdough (*Pediococcus pentosaceus* KTU05-7, KTU05-9 and KTU05-6), increases the redness and improves the sensory properties of pork meat. In addition, BA concentrations in fermented meat samples were far below the levels considered to cause a health risk. Finally, selected bread sourdough strains (*Pediococcus*) can be recommended as technological starters for the pre-treatment of pork meat to improve its quality and safety characteristics.

#### 2.2.3. Sourdough LAB–Plant-Based Bioproducts for Increasing the Functional Value of Meat Products

Nowadays, increasing consumer demand for added-value meat products has led to the creation of new meat product formulations, which include natural, in most cases plant-based, ingredients. The application of bioproducts based on lacto-fermented tomato powder, to increase the biosafety and functional value of meat products, has been reported [65]. During this study, the influence of fermentation on the characteristics of tomato powder was evaluated, and the influence of fermented tomato powder on the quality parameters of ready-to-cook minced pork meat products was established. The main antioxidant in tomatoes is lycopene. Use of the LAB starters *Pediococcus pentosaceus* and *Lactobacillus sakei*, which were previously isolated from spontaneously fermented cereals, increased the β-carotene and lycopene concentrations in tomato powder. The lycopene and β-carotene content in ready-to-cook minced pork meat products was proportional to the amount of fermented tomato powder added. Finally, it was concluded that tomato powder fermented with selected sourdough LAB can be recommended as a colouring and functional ingredient (source of lycopene) in the preparation of ready-to-cook minced pork meat products.

Another plant which receives attention nowadays is *Helianthus tuberosus* L. The main carbohydrate in *H. tuberosus* is inulin; this plant is also a good source of macro- and microelements [66]. Taking into consideration its beneficial properties for human health, due to its specific chemical composition, *H. tuberosus* fermented with sourdough LAB (*P. acidilactici*, *P. pentosaceus*, *L. sakei*) bioproducts has been suggested for use in the preparation of ready-to-cook minced pork meat products [67]. It was found that the fermented *H. tuberosus* additives reduced the undesirable microorganism count and BA concentration in ready-to-cook minced pork meat products. In addition to safety parameters, *H. tuberosus* fermented with sourdough LAB bioproducts led to the formation of specific volatile compounds, which improved the sensory properties of ready-to-cook minced pork meat products.

Savoury plants (*Satureja montana* L.) are associated with compounds possessing antimicrobial properties: carvacrol, terpinen-4-ol, thymol, and linalool [68,69,70]. To reduce the concentration of *S. montana* compounds (due to the very intense flavour, it is not possible to include high concentrations in main food formulations) in ready-to-cook minced pork meat products, combinations of *S. montana* and sourdough LAB possessing antimicrobial properties were tested. Before use, *S. montana* was fermented in both solid-state and submerged conditions with the antimicrobial technological starters [71]. It was established that the viability of LAB in fermented *S. montana* medium depended significantly on the type of fermentation (submerged or solid state). Supplementation of ready-to-cook minced pork meat products with *S. montana* plants fermented in solid-state conditions reduced the growth of mesophilic bacteria, and the highest antimicrobial activity was shown for bioproducts fermented in solid state with the strain *Pediococcus pentosaceus* KTU05-7. Finally, the supplementation of meat products with the combination of *S. montana* and selected sourdough LAB could be a new approach and good alternative for the preparation of ready-to-cook minced pork meat products to prevent meat discoloration and increase the shelf life and concentration of phenolic compounds. In addition, ready-to-cook minced pork meat products incorporating fermented *S. montana* plants showed higher overall acceptability.

The meat produced from different animal species has different sensory properties, some having a specific flavour or taste that is undesirable for consumers. Usually, lamb meat has specific sensory properties inherent to lamb meat. For this reason, the main technological treatments of lamb are different methods of cooking, which help to impart good taste, flavour and aroma, nutritional quality, and safety to lamb meat. Despite these benefits, traditional methods induce potential health hazards, e.g., the formation of polyaromatic hydrocarbons, PAHs, and acrylamide [72]. For this reason, new strategies are sought to improve the lamb meat technological process to obtain safer products. It has been reported that a *Lactobacillus plantarum* strain isolated from bread sourdough, *Thymus vulgaris* essential oil (at a concentration of 0.1% *v*/*v*), and their combination can be used for the pre-treatment of lamb meat [73]. The pre-treatments reduced mould and yeast, as well as total enterobacteria numbers, in lamb meat samples. In addition, the lamb meat colour showed higher acceptability for consumers, in comparison with non-pre-treated lamb meat samples.

Finally, there are many possibilities for the preparation of fermented meat products; selected bread sourdough LAB, which are characterised by GRAS status, could be used as technological starters in meat fermentation processes. However, the final product’s safety characteristics must be taken into consideration, just to ensure formation of the lowest concentration of BAs. Combinations of LAB and plant-based compounds or ingredients could be very promising for the preparation of added-value meat products. In addition, using different antimicrobial compounds, it is possible to promote the effectiveness of the antimicrobial properties and reduce the dose of each compound. Despite the formation of undesirable compounds during the technological process being unavoidable, specific selected solutions may be encouraged to mitigate these issues and limit the formation of toxic compounds to non-toxic concentrations.

### 2.3. Application of Sourdough LAB in the Preparation of Dairy Products

Sourdough LAB possess different properties to ferment carbohydrates and, despite being isolated from fermented cereals, they can show good properties for fermenting lactose. For this reason, they can be used for the preparation of dairy products. Nowadays, consumers are looking for added-value foods, and goat milk cheeses have become popular recently due to their nutritional properties. However, due to their specific sensory characteristics, as well as a short shelf life, consumers do not choose these products. To increase the popularity of these unripened goat milk cheese products showing many benefits for consumers’ health, new strategies are sought for increasing their overall acceptability and prolonging their shelf life. It has been reported that combinations of *Ocimum basilicum* and sourdough LAB such as *Lactobacillus plantarum* LUHS135, *L. paracasei* LUHS244, *Pediococcus pentosaceus* LUHS100, *P. acidilactici* LUHS29, and *L. brevis* LUHS140 immobilised in agar can be used to improve the characteristics of goat cheese [74]. Sourdough LAB and fresh *O. basilicum* plant combinations showed desirable characteristics for the preparation of goat cheese: low pH, a high number of viable LAB, and a high total phenolic compound concentration. Additionally, different LAB included in the LAB–plant-based combinations showed different technological properties, which led to different properties of the cheese. Finally, the authors stated that the immobilisation increases LAB viability in fresh goat milk cheese, which leads to a reduction in enterobacterial and mould/yeast contamination during storage and increases the overall acceptability of the product. Therefore, the developed bioproducts combining selected sourdough LAB (LUHS135, LUHS244, and LUHS140) and plants can be recommended for preparing fresh goat milk cheese with an extended shelf life and high overall acceptability.

A short shelf life is also considered the greatest problem of unripened curd cheeses prepared from cow milk. Mozuriene et al. reported the effect of savoury plants fermented with sourdough LAB on the quality parameters of unripened curd cheese [75]. For the preparation of unripened curd cheese, bacteriocin-producing sourdough LAB strains were selected. Savoury plants (*Satureja montana* and *Rhaponticum carthamoides*) *were fermented with selected sourdough LAB strains* and the prepared bioproducts were used for the preparation of unripened curd cheese. The bioproducts reduced the pH and increased the total titratable acidity and the content of thymol, carvacrol, and *p*-cymene, as well as the overall acceptability of cheese. In addition, bioproducts prepared with *Lactobacillus sakei* decreased the concentration of BAs in cheese, in comparison with unfermented *S. montana* additives.

### 2.4. Application of Sourdough LAB for the Valorisation of by-Products, including Toxin-Degradation Properties

In addition to all their possible uses as food ingredients, sourdough LAB can be used for the valorisation of various food industry by-products, separately and in combination with other pre-treatments, i.e., extrusion and (or) ultrasonication [76,77,78,79].

Despite wheat being the most popular crop in the world, its by-products (bran) have not, till now, been recovered efficiently enough. Due to their specific technological properties, they cannot be incorporated in the main food formulations in high concentrations, and the most popular utilisation of wheat bran is as a low nutritional value feed stock for animal nutrition. However, wheat bran has many health benefits, including dietary fibre, phytoestrogens, etc. [80]. Another challenge associated with the use of wheat bran is its contamination with undesirable microorganisms, as well as with mycotoxins. For this reason, in creating valorisation strategies, these safety issues must be taken into consideration. A new wheat bran valorisation approach has been suggested, combining the processes of extrusion and fermentation with *Lactobacillus plantarum* and *L. uvarum* strains previously isolated from bread sourdough [78]. During this study, the influence of different treatments on BA formation, mycotoxin concentration, and other physico-chemical parameters was analysed. It was established that, in addition to improved nutritional characteristics (higher free amino acid content, lower *trans* fat concentration, etc.), the combination of extrusion and fermentation with selected sourdough LAB strains reduced the mycotoxin and BA concentration in wheat bran. It was confirmed that selected extrusion parameters for pre-treatment of wheat bran and, further, fermenting the extruded wheat bran with selected sourdough LAB starters could be a prospective innovative pre-treatment for the valorisation of wheat by-products, potentially enhancing their safety characteristics and improving their nutritional characteristics.

It has also been reported that the combination of low-frequency ultrasonication and fermentation with *Lactobacillus casei* strain LUHS210 (previously isolated from bread sourdough) can be used for the valorisation of by-products from making plant-based beverages (rice, soy, almond, coconut and oat) [76]. Despite many challenges being associated with by-product valorisation, in addition to desirable technological properties, the most important thing is to ensure the biological and chemical safety of the end products. It was established that the combination of ultrasonication and fermentation increases the biosafety of the tested by-products. Ultrasonication and fermentation with selected sourdough starter reduces the deoxynivalenol concentration in soy beverage by-products. However, after fermentation, 15-acetyldeoxynivalenol was formed in most of the tested by-product samples, the exceptions being those from soy by-products. Additionally, the lowest total BA concentration was found in fermented rice by-products and ultrasonicated coconut by-products. It was concluded that the pre-treatments used could be promising for the valorisation of by-products from the plant-based drinks industry; however, specific technological parameters must be selected, in accordance with specific by-products.

Bartkiene et al. [77] reported changes in the bioactive compounds (phenolic compounds, dietary fibre, lignans, alkylresorcinols, BAs, crude protein, crude fat, fatty acids, free amino acids) in barley industry by-products during submerged and solid-state fermentation with a *Pediococcus acidilactici* strain isolated from bread sourdough. Both fermentation conditions reduced the crude protein content in barley industry by-products and, in contrast, increased the dietary fibre content. Additionally, fermentation had a significant influence on the fatty acid profile (increasing the content of oleic, arachidic, eicosadienoic, behenic, and lignoceric fatty acids) and reduced the BA content in barley industry by-products, in comparison with unfermented samples.

Possible applications of sourdough LAB for the valorisation of by-products are shown in Table 2.

Finally, reported data about the characteristics of valorised by-products are very important because further recommendations for the valorisation of by-products and their possible uses in the food, nutraceutical, or feed industries can be suggested according to the formation of specific compounds during the fermentation process, safety parameters, and the changes of functional properties.

### 2.5. Application of Sourdough LAB for Higher-Value Food and Nutraceutical Formulations

Sourdough LAB could be very promising ingredients for the preparation of higher-value products, because fermentation with selected LAB strains leads to better sensory properties and improves the technological properties of plant-based proteins. Today, when problems associated with climate changes are a very important issue, in most countries, current protein production has started to focus on plant-based protein sources. Despite there being open discussions worldwide about the sustainability of animal proteins, the possibility of including more plant protein sources in traditional or new food formulations becomes very attractive due to the specific functional properties of plant proteins. From this point of view, plant protein becomes a very promising environmentally friendly alternative to animal-based food ingredients. The crops that can be utilised for protein preparation include beans, canola, hemp, linseed, white maize, oats, peas, potato, walnut, alfalfa (lucerne), barley, wheat, etc. However, the main challenges in utilising most plant-based protein sources are poor functionality and unacceptable sensory properties. The possible application of sourdough LAB possessing antimicrobial properties in the preparation of hemp seed (*Cannabis sativa* L.)-based beverages in emulsion form has been reported [81]. In addition to technological and functional characteristics, the BA content and antimicrobial properties of the prepared emulsions were analysed. Before fermentation, ultrasonication of hemp seed was applied as a pre-treatment to reduce microbial contamination of the raw material. It was established that, despite the pure sourdough LAB inhibiting a broad variety of pathogenic and opportunistic strains, the antimicrobial activity of prepared hemp seed-based emulsions was very low. It was suggested that treatment with selected sourdough LAB can be recommended for the preparation of stable emulsions, and that, by selecting appropriate LAB strains, it is possible to prepare hemp seed beverages with very good sensory properties.

Another study reported a technology for the preparation of pea snacks, which was based on solid-state and submerged fermentation of peas with two sourdough LAB strains (*Lactobacillus casei* LUHS210 and *L. uvarum* LUHS245) [82]. Ultrasonication was applied as a pre-treatment for the raw material (peas), and the biosafety parameters and BA concentration of the products were analysed to ensure the safety of the technologies used. Additionally, during this study, a different salt content was tested for the preparation of proteinaceous snacks. It was established that ultrasonication in combination with fermentation with selected sourdough strains reduced the number of enterobacteria in peas, and no yeast or mould was found in fermented peas. The fermentation method (solid state or submerged), LAB strain used, and ultrasonication had a significant influence on the texture of the final product. Despite the predominant BAs in treated peas being phenylethylamine and spermidine, a reduction in these compounds was observed in fermented samples. Finally, it was suggested that the pea snacks most acceptable to consumers could be obtained by pre-treating peas with ultrasonication and fermenting them with selected LAB strains with the addition of 1.0 g/100 g salt.

To improve the traditional diet, the use of food enriched with functional ingredients has become very attractive. Functional compounds can be suggested in ‘dose’ form, such as tablets and capsules. However, for most consumers, these forms are associated with medical treatment. For this reason, to increase consumption of the functional ingredients, they alternatively can be incorporated into food formulations, for example, in the form of gummy candies. A study reported the development of functional gummy candies based on lupine protein concentrates lacto-fermented with sourdough LAB, *Citrus paradisi* essential oil, and xylitol [83]. To improve the functional value of lupine protein, submerged fermentation with *Lactobacillus sakei* was used. Prepared lupine seed protein concentrate contained 90.1% protein, and protein digestibility in vitro was 89.94%. The concentration of the isoflavone genistein in lupine protein isolate was 30.93 μg g^−1^, and these samples showed the lowest trypsin inhibitor activity, in comparison with lupine protein isolates, which were prepared using other LAB strains. The authors suggested that a gummy candy formulation containing xylitol, ascorbic acid, *C. paradisi* essential oil (up to 0.2%), and fermented lupine protein concentrate (up to 13.0%) produces candies with good texture, high overall acceptability, and containing desirable functional compounds.

Sourdough LAB can be used to produce exclusive higher-value products based on compounds of different origin. For example, nutraceuticals in gummy candy form possessing antimicrobial properties can be prepared using combinations of LAB strains (*Lactobacillus plantarum* LUHS135 and *L. paracasei* LUHS244), bovine colostrum, and essential oils (*C. reticulata* L., *Eugenia caryophyllata*, *C. paradisi* L., *Thymus vulgaris* and their emulsions (12%)) [16]. The antimicrobial activity of the tested ingredients and their combinations against pathogenic bacteria strains (*Pseudomonas aeruginosa*, *Proteus mirabilis*, *Escherichia coli*, *Salmonella enterica*, *Staphylococcus aureus*, *Enterococcus faecalis*, *Streptococcus mutans*) were investigated, and the best formulation of components for the preparation of antimicrobial nutraceuticals in gummy candy form was suggested as bovine colostrum fermented with *L. paracasei* LUHS244 (up to 3%) in combination with *T. vulgaris* or *E. caryophyllata* essential oil (up to 0.2%), which inhibited growth of all the tested pathogenic microorganisms (except *P. aeruginosa*).

Additionally, to produce higher-value products, sourdough LAB can be used for the conversion of dairy industry by-products (for example, milk permeate) to additional value compounds [84,85]. Fermented milk permeate is a good source of galactooligosaccharides and viable LAB, which possess desirable antimicrobial properties. Additionally, the incorporation of psyllium husk (a source of desirable hydrocolloids) and apple by-products (a source of phenolic compounds) in this bioconverted dairy industry by-product can lead to the development of high-value food formulas. In addition to nutraceuticals in gummy candy form, added-value beverages can be produced by converting food industry by-products (milk permeate, wheat bran, fruit, and berry industry by-products, etc.) with the selected sourdough LAB. In another study [86], the main ingredients used for the preparation of added-value beverages were fermented milk permeate (containing galactooligosaccharides), extruded and fermented wheat bran (containing 8.79 log_10_ CFU g^−1^ viable LAB strains showing antimicrobial properties), and different fruit/berry by-products (as a source of antioxidants). The functional properties of the beverages were proved by evaluating their antimicrobial and antioxidant properties, as well as the viable LAB count during storage. Desirable changes in extruded and fermented wheat bran were obtained: fermentation reduced the sugar concentration and pH in samples with predominant lactic acid isomer L(+). In addition, the viable LAB count in the substrate was higher than 6.0 log_10_ CFU g^−1^, and no enterobacteria remained. Fruit and berry industry by-products showed desirable antimicrobial activity: elderberry, sea buckthorn, blueberries, and raspberries inhibited 2, 3, 5, and 7 of the 10 pathogens tested [84]. By comparing the samples prepared with the addition of fermented wheat bran with those prepared with fermented wheat bran and fruit and berry industry by-products, it was observed that most fruit and berry by-products increased the total phenolic compound content in beverages. Finally, the newly developed nutraceutical beverages possessed desirable antimicrobial and antioxidant properties, while being prepared in a sustainable and environmentally friendly manner. Additionally, LAB in combination with bovine colostrum, apple by-products, and essential oils can favourably alter the host immune system and gut microbiota in a swine model [84,87].

## 3. Sourdough LAB and Their Prospective Antimicrobial Combinations with Plant- and Animal-Based Ingredients

It has been reported that an effective alternative to antibiotics, which can help to reduce the occurrence of antibiotic-resistant *Salmonella* strains, is a composition containing 1.0% thyme essential oil and the following sourdough LAB strains: *Lactobacillus plantarum* LUHS122, *Enterococcus pseudoavium* LUHS242, *L. casei* LUHS210, *L. paracasei* LUHS244, *L. plantarum* LUHS135, *L. coryniformis* LUHS71, and *L. uvarum* LUHS245 [88]. However, most LAB strains are sensitive to environmental conditions, and it should be pointed out that most of the above-mentioned LAB strains were inhibited by thyme essential oil at concentrations of 0.5% and 1.0%, except for LUHS122, LUHS210, and LUHS245. However, it can be noted that the agents responsible for the inhibition of *Salmonella* are not the viable LAB strains but rather their metabolites, and further studies are needed to identify which metabolites are the most important [88]. Additionally, a synergistic mechanism of a combination of *L. plantarum* LUHS135 and Baltic Sea macro-algae (*Ulva intestinalis*, *Cladophora rupestris* and *Furcellaria lumbricalis*) showed a broader spectrum of pathogen inhibition, in comparison with algae alone [89]; these findings could lead to broader application of macro-algae biomass for various purposes.

Finally, combinations of plant- or animal-based ingredients with selected sourdough LAB are very promising for the development of new added-value products.

## 4. Sourdough LAB as Biological Agents for Modelling the Immune System and Digestion Microbiota

In vivo studies with a piglet model have shown that supplementary feeding with a nutraceutical formula based on sourdough LAB in combination with bovine colostrum, apple by-products, and essential oils results in a statistically significant decrease in the proportions of T cytotoxic and double-positive (CD4^+^CD8^+low^) cells within the CD3^+^ cell population at 28 DPI, compared to the beginning of the experiment (0 DPI). Conversely, a significant increase in the proportions of B cells (accompanied by an increase in IgG concentration) and macrophage/monocyte cells was observed within the viable cell population at 28 DPI, compared to the beginning of the experiments. Furthermore, changes in the bacterial composition of the gut microbiota in pigs fed with a multicomponent nutraceutical changed significantly, with a 1.78 times higher number of probiotic strains (*Bifidobacterium*, *Streptococcus*, *Faecalibacterium*) at the end of the experiment, compared to control group animals. Finally, the nutraceutical formula based on LAB in combination with bovine colostrum, apple by-products, and essential oils had a positive effect on the changes of gut microbiota by facilitating an increase in probiotic bacterial strains, and possible anti-inflammatory properties were developed.

In addition to nutraceuticals and food, fermentation technology based on sourdough LAB is very promising in livestock production [90,91,92,93,94,95,96]. A combination of microbial starters, previously isolated from spontaneously fermented sourdough—*Lactobacillus uvarum* LUHS245, *L. casei* LUHS210, *Pediococcus acidilactici* LUHS29, and *P. pentosaceus* LUHS183—was used for fermentation of piglet feed; fermented feed parameters, as well as the influence of fermented feed on piglets’ faecal microbiota, health, and growth performance, were evaluated [97]. Additionally, mycotoxin biotransformation in vivo, including masked mycotoxins, was analysed in feed and piglet faecal samples. The 36-day experiment was conducted using 25-day-old Large White/Norwegian Landrace piglets. Analysis of piglets’ faecal microbiota showed an increased number of probiotic bacteria in the treated group, particularly *Lactobacillus*, when compared with the control group at the end of the experiment. This finding indicates that fermented feed can modify the microbial profile in the gut of pigs. Mycotoxin analysis showed alternariol monomethyl ether and altenuene in the faeces of 61-day-old control piglets and in fermented feed samples. However, no alternariol monomethyl ether was found in the faeces of treated piglets. Fermentation of feed with the novel sourdough LAB combination is a promising means to modulate piglets’ microbiota, which is essential to improve nutrient absorption, growth performance, and health parameters. Finally, the new LAB composition suggests a novel dietary strategy to positively manipulate fermented feed chemicals, biosafety, and the piglet gut microbial ecology, to reduce the use of antimicrobials in pig production and increase the use of local feed stock and the economical effectiveness of the process. Another study with piglets [98], which used LAB strains newly isolated from bread sourdough (*Lactobacillus plantarum* LUHS122, *L. casei* LUHS210, *L. farraginis* LUHS206, *P. acidilactici* LUHS29, *L. plantarum* LUHS135 and *L. uvarum* LUHS245), showed that fermentation had a positive impact on the prevalence of *Lactobacillus* during the post-weaning period of pigs, and vaccination had no negative impact on microbial communities, although a larger amount of *Lactobacillus* was detected in vaccinated groups than in unvaccinated groups. At the end of the experiment, there was a significantly higher LAB count in the faeces of both vaccinated groups compared to unvaccinated groups. Despite there being no significant differences in the average daily gain among the groups, there were significant differences in the feed conversion ratios between groups, and the lowest ammonia emission was found in the group fed with fermented feed. Finally, it was concluded that, by changing from an extruded soya to a cheaper rapeseed meal and applying the fermentation model with the selected LAB combination, it is possible to feed piglets in a more sustainable manner without any undesirable changes in health and growth performance [98].

## 5. Conclusions

As could be seen from this review, there are many applications of sourdough LAB. In addition to their application as pure technological strains in the food and feed industries, taking into consideration the antimicrobial, antifungal, immuno-, and microbiota-modulating properties of these microorganisms, they could be used as valuable ingredients in higher-value food as well as nutraceutical formulations. Additionally, a very promising application of LAB is their use in combination with plant- and/or animal-based ingredients to increase the functional properties of the whole combination due to different mechanisms of action, as well as desirable symbiotic activity. Finally, taking into consideration that sourdough LAB application at an industrial scale is not efficient enough, this review is intended to highlight that bread sourdough is a very promising source of technological microorganisms, which has great potential at an industrial scale. Future prospects for sourdough LAB application are very broad, as they have potential to be used in a wide range of agri-food industries—such as baking, feed and pet food, dairy, meat, nutraceuticals, alcoholic and non-alcoholic beverages, cosmetics, pharmaceuticals, chemicals, etc.

## Figures and Tables

**Table 1:** Possible applications of sourdough lactic acid bacteria (LAB) in bread preparation.

Sourdough LAB Strains	Possible Applications	Reference
*Pediococcus acidilactici* LUHS29	For barley sourdough fermentation and the preparation of higher-value bread	[19]
Combinations of LAB strains: *Pediococcus pentosaceus* LUHS183 and *Leuconostoc mesenteroides* LUHS242, *P. pentosaceus* LUHS183 and *Lactobacillus brevis* LUHS173, *P. pentosaceus* LUHS183 and *Enterococcus pseudoavium* LUHS234, *P. pentosaceus* LUHS183 and *Lactobacillus curvatus* LUHS51, *Lactobacillus plantarum* LUHS135 and *L. curvatus* LUHS51, *L. plantarum* LUHS135 and *P. pentosaceus* LUHS183	For wheat bread quality improving (higher porosity, better sensory properties, lower acrylamide concentration)	[20]
*Lactobacillus coryniformis* LUHS71, *L. curvatus* LUHS51, *L. farraginis* LUHS206 and *Leuconostoc mesenteroides* LUHS225	For wheat bread quality improving (higher porosity, better sensory properties, lower acrylamide concentration); For surface treatment of bread to prolong the shelf life	[22]
*Pediococcus pentosaceus* LUHS183, *P. acidilactici* LUHS29, *Lactobacillus paracasei* LUHS244, *Lactobacillus brevis* LUHS173, *Lactobacillus plantarum* LUHS135 and *Leuconostoc mesenteroides* LUHS242	As antifungal agents against *Aspergillus nidulans*, *Penicillium funiculosum* and *Fusarium poae;* For bread safety improving (lower acrylamide concentration)	[23]
*Lactobacillus plantarum* LUHS135 in combination with savory plants *Thymus vulgaris*, *Carum carvi*, *Origanum vulgare*, *Ocimum basilicum* and *Coriandrum sativum*	For bread safety improving (lower acrylamide concentration)	[24]
*Lacticaseibacillus casei* LUHS210	For almond, coconut and oat drinks by-products valorisation and added-value bread preparation	[25]
*Lactobacillus paracasei* LUHS244	For okara valorisation and added-value bread preparation	[27]

**Table 2:** Possible applications of sourdough LAB for the valorisation of by-products.

Sourdough LAB Strains	Possible Applications	Reference
*Lactobacillus plantarum*, *L. uvarum*	For wheat production by-products valorisation (safety and nutritional characteristics improving)	[78]
*Lactobacillus casei* strain LUHS210	For rice, soy, almond, coconut, and oat drinks production by-products valorisation	[76]
*Pediococcus acidilactici*	For barley industry by-products valorisation	[77]

## Data Availability

Not applicable.
